# Implications of Exceeding the Age and Interventions to Extend Life Purpose: Perspectives From Asian Centenarians

**DOI:** 10.1093/geroni/igaa057.073

**Published:** 2020-12-16

**Authors:** Ad Maulod, Normala Manap, Sasha Rouse, Angelique W M Chan

**Affiliations:** Duke-NUS Medical School, Singapore, Singapore

## Abstract

Centenarians have often been regarded as living paradigms of exceptional longevity yet little is known from their perspective about the purpose, meaning and quality of living longer lives. In Singapore, the number of centenarians has multiplied 30-fold from 50 in 1990 to about 1500 in 2020. Although centenarians are respected as ‘national treasures’ – having witnessed Singapore’s transformation from British colony to global city state, their needs remain invisible in both the healthcare and social sectors. The tendency to romanticize exceptional longevity neglects a deeper understanding of (i) its consequence on the oldest old (85+ years) who may be impacted by severe functional and sensory deficits and (ii) their experience of social isolation in the family and communities. This paper discusses findings based on interviews with 15 Singapore centenarians (100 to 111 years old) and their family carers. Diverse experiences of longevity are shaped by these factors: health status; personal disposition; strength of family and social networks; exposure to adversity and coping resources; spiritual beliefs; role loss; and changes in the lived environment. Appropriate health and psychosocial interventions could have been delivered earlier in the life trajectory to enable better quality of life and continued social engagement. Learning from the challenges (eg. social withdrawal; extensive functional and sensory losses) of existing centenarians contributes to a more precise understanding of how best to harness the productive capacities of our oldest old.

